# Minor Surgery

**Published:** 1912-06

**Authors:** 


**Minor Surgery.** By LEONARD A. BIDWELL, F.R.C.S. Pp. xiv, 265. London: University of London Press. 1911.

This is a useful book, containing a clear and succinct account of minor surgery, and of the application of it to modern methods.

